# Applying missing data methods to routine data: a prospective, population-based register of people with diabetes

**DOI:** 10.1186/1745-6215-14-S1-P113

**Published:** 2013-11-29

**Authors:** Stephanie Read, Sarah Wild, Steff Lewis

**Affiliations:** 1 University of Edinburgh, Edinburgh, UK; 2 Scottish Diabetes Research Network Epidemiology Group, Scotland, UK

## Background

Routinely-collected data could be used to make randomised controlled trials (RCTs) more efficient, either for collection of outcome data or to enhance recruitment. The use of routine data in RCTs has been limited by concerns surrounding data quality, particularly missingness. To exploit these information-rich data sources, it is necessary to identify approaches capable of overcoming high rates of missing data.

## Methods

Using data from a population-based diabetes register linked to mortality records, we compared four methods for handling missing data when investigating the association between body mass index and all-cause mortality in patients with Type 2 diabetes in a retrospective cohort study. Complete case analysis (CCA), population mean imputation (PMI), stochastic imputation (SI) and multiple imputation (MI) methods were applied to handle the missing data. Cox proportional hazard model coefficients for the association between BMI and all-cause mortality were compared for each missing data method.

## Results

Body mass index data were unavailable for 117,048 (54.07%) patients and there were 41,555 deaths among the cohort between 2001 and 2008. Data appeared to be missing at random conditional on year of diagnosis and health status. CCA produced a J-shaped relationship between patient BMI and all-cause mortality, though findings from other approaches indicated that CCA underestimated the survival in this population. Estimates obtained from SI and MI flattened the observed J-shaped curve. However, imputations were based on poor predictions.

## Summary

Different approaches for handling missing data can influence associations and caution is required when using incomplete routine data to improve RCTs.

